# Prefabricated concrete components combination schemes selection based on comprehensive benefits analysis

**DOI:** 10.1371/journal.pone.0288742

**Published:** 2023-07-26

**Authors:** Shuqiang Wang, Zengjiayu Wang, Yuke Ruan

**Affiliations:** School of Civil Engineering, Architecture and Environment, Hubei University of Technology, Wuhan, China; University of Birmingham, UNITED KINGDOM

## Abstract

In prefabricated buildings, there are numerous types of prefabricated components, forming a complex combination of schemes that are difficult to select. Therefore, this article takes prefabricated components combination schemes as the object. By constructing the evaluation index system through four aspects of assembly rate, cost, duration, and carbon footprint, then using the fuzzy gray correlation projection method to evaluate and select. A residential in Wuhan, China, was enlisted to conduct a case study to show the application of the proposed method. Results indicate that among the six choices, the L scheme is optimal, and the selection order of the prefabricated components in different scenarios is ranked. The results reveal that the method has good applicability, simultaneously provides a reasonable and effective reference for each participant of the assembled building when making scheme comparison, and also provides a new method for the evaluation study of prefabricated component combination schemes.

## Introduction

With the development of the economy, assembled construction has gradually become the construction method favored by the construction industry with its advantages of high productivity, short construction cycle, less construction waste, resource-saving, and low energy consumption. Along with the continuous promotion of government policies and the advantages of prefabrication, the demand for prefabricated components, as an important part of prefabricated buildings, will continue to rise in the future [1–3]. In addition, prefabricated components’ type and the way they are combined have a prerequisite effect on the assembly cost. How to choose the prefabricated components combination scheme becomes a cardinal discussion issue.

At present, there are several approaches to the selection of prefabricated plans in general: statistical, quantitative analysis, etc. Jin et al. [4] through data statistics and generalization, the three requirements of low, medium, and high prefabrication rates are distinguished, and the principles and schemes of prefabricated structural components selection are summarized. Gu et al. [5] took the actual project design scheme as an example, and through the analysis of the cost and duration of multiple options, the conclusion was obtained that in a similar large shopping center-type commercial project in Zhejiang, China, the option of prefabrication of vertical component is preferred. However, further research is needed to select the appropriate combination of prefabricated components based on the consideration of cost, quality, duration, and environment.

The selection of the optimal prefabricated components scheme can be analyzed by evaluating its comprehensive benefits. For assembled buildings, the comprehensive benefit refers to the overall benefit and comprehensive impact of the combination of economic, social, environmental, and technological benefits generated by assembling in all aspects of the whole life cycle [6]. Different prefabricated components combination schemes have different impacts on the overall benefits of assembled buildings, as well as different impacts on cost, schedule, and quality. Therefore, the evaluation and selection of prefabricated components combination schemes for assembled buildings have an obvious significance. Nevertheless, the research on it is still in the exploration stage, the methods used are more subjective, and the selection of prefabricated components and the selection of combination schemes are mostly considered from one aspect, which is not comprehensive sufficiently [7].

The main purposes of this study include the following:

1. By quantifying the evaluation indexes of the prefabricated components combination schemes, it provides data support for the subsequent selection of a suitable combination scheme.
2. Constructing a reasonable combination of scheme decision-making methods to achieve the selection of scheme with the optimal comprehensive benefits and providing a scientific and efficient selection method for the decision-makers.

Consequently, in this paper, based on commonly used prefabricated components from the perspective of comprehensive benefits, a variety of components combination schemes are formed based on the principle of permutation and combination, and prefabricated components combination schemes are selected by the fuzzy gray correlation projection method.

## Literature review

### Prefabricated components combination schemes in previous studies

The selection of prefabricated components combination schemes will not only affect the safety of the assembled building, but also be able to control the cost, environment, and other aspects.

Many scholars have studied the impact of the selection of different prefabricated components on the cost. Chen and Huang [8] compared the difference in unit cost under different prefabrication rates and analyzed the effect of different prefabrication rates on the construction cost of assembled buildings. The results showed that the use of prefabricated laminated slabs, prefabricated stairs, and prefabricated non-load bearing walls in the shear wall structure of assembled buildings would not increase the cost significantly, and also provided a theoretical reference for structural design. Hong et al. [9] used a cost-benefit analysis framework to show that concrete and steel costs account for the largest share of typical precast components, followed by labor and transportation. Samani et al. [10] compared the life-cycle costs of the two building types by NPV and showed significant differences in the costs in different regions. Xu and Wu [11] from the perspective of building components, the cost of assembled building and cast-in-place building components are compared and analyzed in terms of labor, material, and machine under the engineering bill pricing mode, and the selection order of assembled building components is obtained. Xu et al. [12] adjusted the ratio of each prefabricated component and analyzed the relationship between prefabrication rate and cost of prefabricated components of assembled buildings, showing when the prefabrication rate was 15%, 20%, 25%, 28%, and 30%, the combination with the least incremental cost of assembled buildings was prefabricated laminated slabs and laminated beams.

Additionally, the selection of different prefabricated components and combination options has different environmental impacts. Hao et al. [13] quantified the percentage reduction of construction waste and found that the widespread use of prefabricated components reduces the cost of construction waste management in China while mitigating the global construction waste impact on the environment and society. Li et al. [14] based on the Life Cycle Assessment (LCA) method, compared the carbon footprint of precast and cast-in-place components, and the results showed precast concrete components can reduce the carbon footprint, especially in the materialization phase that generates most of the carbon emissions. Wang et al. [15] investigated the differences in life-cycle environmental impacts between three different types of subfloors, using the SimaPro, with pre-slabs being the second largest contributor to environmental impacts. Naji et al. [16] coupled TRNSYS and jEPlus + EA for multi-objective optimization of the building envelope, and prefabricated components were selected for different climate zones. Wang and Sinha [17] compared nine scenarios with prefabrication rates ranging from 6% to 96%, and the results indicated an increase in the water footprint when the prefabrication rate increases, which also leads to changes in the energy footprint, carbon footprint, and terrestrial ecotoxicity.

Most researchers in the above-mentioned studies analyzed the prefabricated components combination schemes in terms of individual influence conditions. How can the most suitable prefabricated components combination scheme be selected based on satisfying several conditions is worth attention, but there are fewer related studies. To fill this void, this paper aims to study the combination of the prefabricated components, get the selection order of the prefabricated component combination scheme, and find the most suitable combination scheme, which is important to achieve sustainable economic development.

### Evaluation methods in previous studies

Comprehensive evaluation is a systematic and complex task and is one of the most important means to know, understand and influence things [18].

When evaluating the assembled buildings, comprehensive evaluation methods at home and abroad include principal component analysis, gray correlation projection method, fuzzy evaluation method, etc. Bian et al. [19] used principal component analysis to rank the order of importance of factors that constrain the development of assembled buildings and proposed countermeasures to promote the development of assembled buildings. Chen et al. [20] through the risk assessment model of construction quality based on interval hierarchy analysis and fuzzy theory assess the risk of construction quality of assembled buildings. Yang et al. [21] combined grey system theory and vector projection principle to establish a comprehensive model for investment decision-making in construction projects. Luo et al. [22] established a site selection index system for precast concrete plants based on the general site selection method using fuzzy hierarchical analysis, which helps construct manufacturers make investment decisions by simulating the actual precast plant site selection.

The commonly used evaluation methods are too subjective on the one hand, requiring the judges to determine the index weights based on their own experience, and on the other hand, the algorithms are complicated and difficult to operate, unable to be applied to actual projects, meanwhile, the incompleteness and ambiguity of information are rarely considered at the same time. In Table 1, the characteristics and drawbacks of the commonly used methods are summarized.

**Table 1:** Evaluation method analysis.

Method	Characteristics	Drawbacks
**Principal component analysis**	Eliminate the influence of correlation between evaluation indicators and classify evaluation objects.	Cannot effectively eliminate information overlap.
**Fuzzy evaluation method**	Be able to accurately quantify fuzzy information and make reasonable quantitative evaluations.	The determination of the weight of each factor is somewhat subjective.
**Grey Relation Analysis**	No requirement for sample size, simple calculation process, and different results for different methods of correlation calculation.	The choice of indicators has a significant impact on the judging results.
**Vector projection method**	Transformation of high-dimensional data into low-dimensional data utilizing projection, thus reducing the computational difficulty.	Difficult to find suitable and ideal reference objects.
**Gray correlation projection method**	urther strengthening important indicator weighting factors.	Lack of consideration of the distance from the decision solution to the ideal inferior solution.

The evaluation object of this paper is the prefabricated components combination scheme, which is a complex multi-objective and multi-criteria decision-making evaluation problem. Combining the analysis of each method in the above table, using the gray correlation projection method, the cosine values between different solutions and the ideal superior and inferior solutions were calculated. The larger the cosine value, the closer the alternate solution is to the ideal solution, and vice versa. However, this method does not consider the distance from the decision solution to the ideal inferior solution, as well as due to the cosine value change may lead to inaccurate results. In lieu, the fuzzy evaluation method uses fuzzy theory to calculate the superiority of the decision solution to the ideal superior and inferior solutions to fill the shortcomings of the method. Each combination alternative can be described in a simple mathematical form, combining the fuzzy gray correlation projection method is easy to apply, reasonable, easy to understand, has a solid mathematical basis, strong operability, etc. It can analyze the relevance of each index in a comprehensive and specific way, avoiding the bias induced by comparing only the index values of individual factors of each scheme, making the evaluation results more accurate and scientific. Therefore, it was adopted for the preferential selection of prefabricated component combination schemes for assembled buildings.

In general, research on prefabricated components has been carried out from different perspectives and is relatively mature, but there is a lack of an effective method to select the prefabricated components combination scheme in the current research. By reviewing domestic and foreign literature, using fuzzy gray correlation projection method is applied to the evaluation preference of assembly building prefabricated component combination scheme. A scientific and objective evaluation method is used to evaluate and analyze the combination scheme, so as to correctly deal with the selection problem of a multi-objective scheme. It lays the model foundation for subsequent case analysis of selecting prefabricated component combination schemes, making the decision results more realistic and credible. It also provides a reference and theoretical basis for the pre-decision of prefabricated buildings, thus promoting its development.

## Method

**Fig 1:**
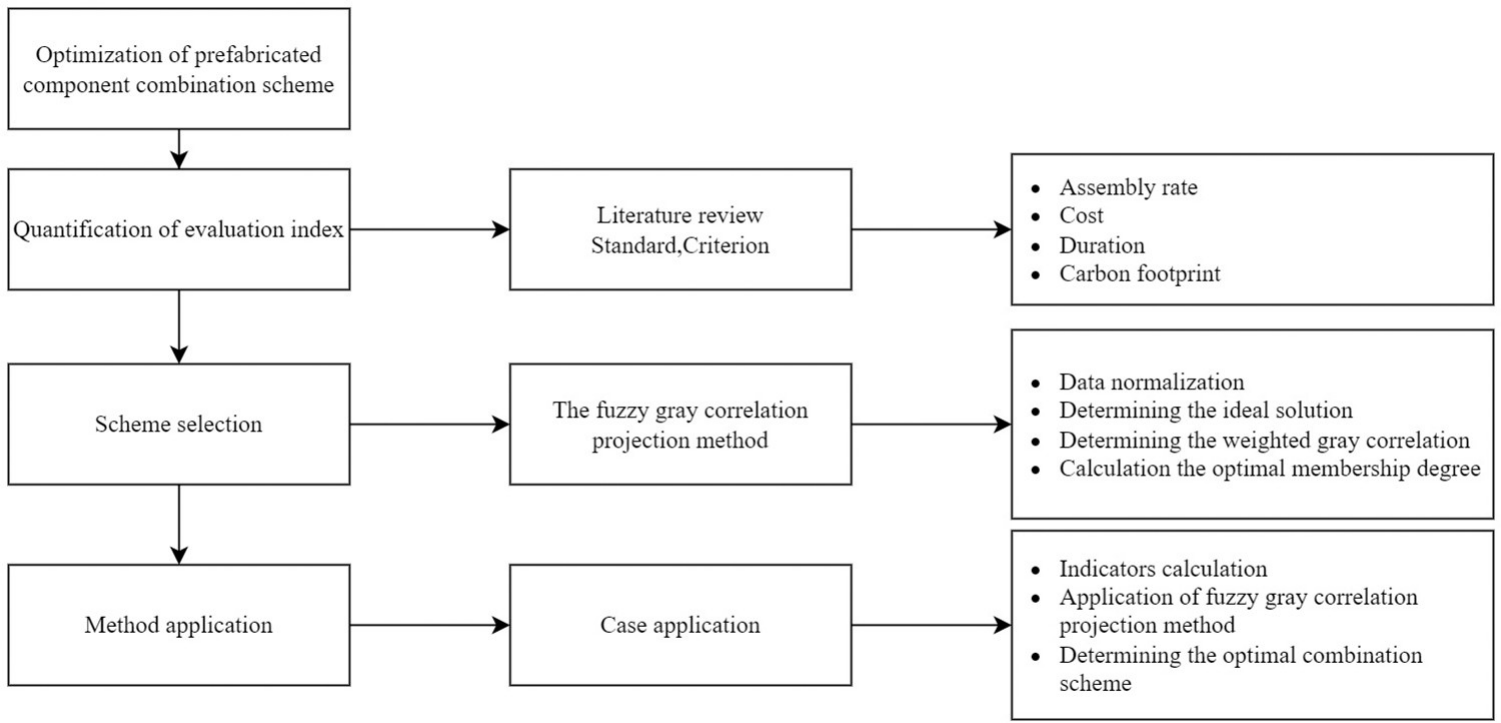
Selection method of combination schemes.

**Fig 2:**
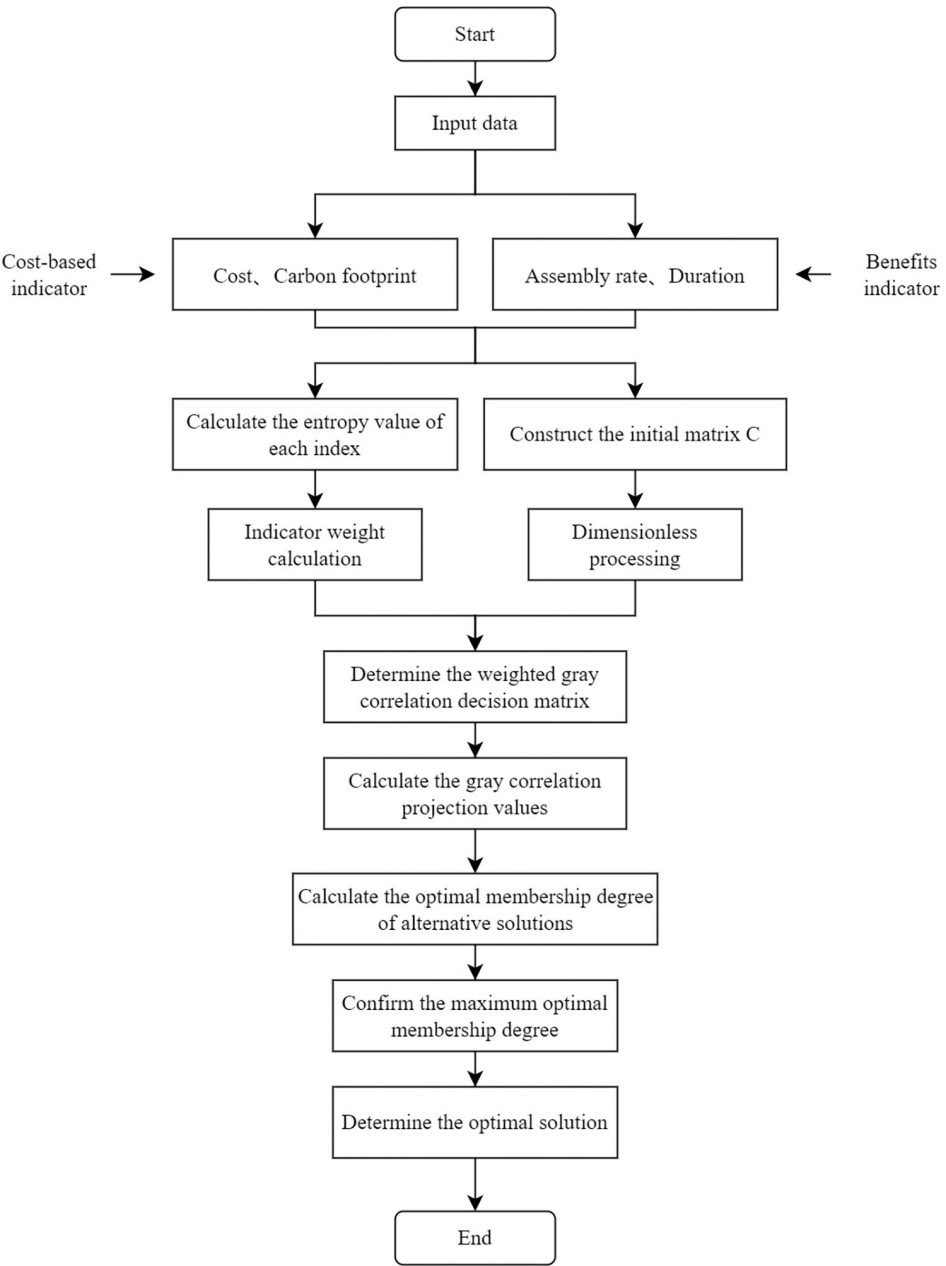
The flowchart of the selection method.

In this paper, based on relevant researches, a quantitative evaluation index system is constructed in terms of four aspects: assembly rate, cost, duration, and carbon emission, and the representativeness and comparability of different indexes are considered by integrating existing data and literature. Eventually, the fuzzy gray correlation projection method is used for scheme decision-making. To achieve the objectives of this study, the method framework is shown in Fig 1. The specific steps for the selection of prefabricated component combination schemes based on the fuzzy gray correlation projection method were shown in Fig 2.

### Quantitative evaluation index system

#### Assembly rate

Assembly rate is a standard to measure the degree of assembly of industrialized buildings using prefabricated components. By now, the government has taken assembled buildings into the scope of construction project evaluation, and there are explicit and reasonable requirements for the assembly rate. For different buildings’ heights and structural systems, suitable assembly rates should be selected, too high assembly rates make the cost of construction higher, while too low assembly rates will have an impact on the output of construction enterprises and prefabricated component factories, so it is particularly important to select suitable assembly rates in the prefabricated components combination schemes selection.

According to the “Standard for assessment of prefabricated building”, the assembly rate is calculated as follows.

$$P=\frac{Q_{1}+Q_{2}+Q_{3}}{100-Q_{4}}*100\%$$ (1)

Where P refers to the assembly rate, *Q*_1_, *Q*_2_, and *Q*_3_ refer to the actual score values of the main structure, maintenance wall and internal partition wall, and decoration and equipment piping respectively; *Q*_4_ refers to the sum of the scores of the lacking evaluation items in the evaluation items.

**Cost.** The high investment in the initial phase of assembled buildings and the majority of companies do not have an intuitive understanding of the economic benefits it brings have led many companies to turn away from it, so research on the cost indices of assembled buildings is necessary.

Over comparative analysis of the literature, it is concluded that the cost of civil engineering accounts for more than 80% of the project cost, including direct costs, indirect costs, taxes, and profits. The indirect costs, profits, and taxes are calculated based on direct costs. The direct costs of the cast-in-place building are composed of labor cost, material cost, measure expense, and construction machinery use fee. Moreover, the assembled building also includes the production cost, transportation cost, and installation charge of prefabricated components. Hence, this paper selects labor cost, material cost, construction machinery use fee, transportation cost, and measure expense to analyze the cost indices.

Labor cost

The labor cost includes the labor cost for casting-in-place, production of prefabricated components, and later on-site assembly, calculated as follows.

$$C_{p}=C_{cp}+C_{pp}+C_{ip}$$ (2)

$$C_{p}=\sum_{a=1}^{n}M_{1a}\times V_{a}+\sum_{i=1}^{n}N_{2i}\times V_{i}\times Q_{k}+\sum_{i=1}^{n}N_{1i}\times V_{i}\times Q_{l}$$ (3)

Where *C*_*p*_ refers to the labor cost of each phase in the project; *C*_*cp*_, *C*_*pp*_, and *C*_*ip*_ refer to the labor cost of the cast-in-place phase, the production of precast components phase, and the on-site assembly of precast components, respectively; *M*_1*a*_ refers to the labor cost per unit volume of cast-in-place part a; *V*_*a*_ refers to the volume of cast-in-place part a; *V*_*i*_ refers to the total volume of i^th^ precast components; *N*_1*i*_ and *N*_2*i*_ represent the total man-hours for the installation and production of prefabricated components of the i type per unit volume; *Q*_*k*_ and *Q*_*l*_ refer to the market unit price of man-hours of production and installation workers.

Material cost

Material cost includes the material cost for the cast-in-place portion, production of prefabricated components, and on-site assembly, calculated as follows:

$$C_{m}=C_{cm}+C_{pm}+C_{im}$$ (4)

$$C_{m}=\sum_{a=1}^{n}M_{2a}\times V_{a}+\sum_{i=1}^{n}C_{i}\times D_{l}\times V_{i}+\sum_{k=1}^{n}N_{ik}+D_{k}+V_{i}$$ (5)

Where *C*_*m*_ refers to the material cost of each stage in the project; *C*_*cm*_, *C*_*pm*_, and *C*_*im*_ refer to the material cost of cast-in-place part, production of the precast part, and on-site assembly respectively; M2a refers to the material cost of cast-in-place part a; *C*_*i*_ refers to the market unit price of material required for the production of ith precast part; *D*_*i*_ refers to the quantity of material required for the production of ith precast part; *N*_*ik*_ refers to the cost of material of class k when installing ith precast part; *D*_*k*_ refers to *k*^*th*^ material’s market unit price.

Construction machinery use fee

Including the fee of the cast-in-place part, the production of prefabricated components part, and the on-site assembly part, calculated as follows:

$$C_{n}=C_{cn}+C_{pn}+C_{in}$$ (6)

$$C_{m}=\sum_{a=1}^{n}M_{3a}\times V_{a}+\sum_{l=1}^{n}E_{il}\times V_{i}\times P_{l}+\sum_{f=1}^{n}E_{if}+V_{i}+P_{f}$$ (7)

Where *C*_*n*_ refers to the fee of using machines in each stage of the project; *C*_*cn*_, *C*_*pn*_, and *C*_*in*_ refer to the fee of machines for the casting-in-place part, production of prefabricated components, and on-site assembly; *M*_3*a*_ refers to the fee of machines for casting-in-place part a; *E*_*il*_ refers to the shift of l-type machines consumed in the production of i-type prefabricated components; *E*_*if*_ refers to the shift of f-type machines consumed in the installation of i-type prefabricated components; *P*_*l*_ refers to the unit price of l-type machinery shift; *P*_*f*_ refers to the unit price of f-type machinery shift.

Measure expense

According to the characteristics of prefabricated components installed on-site, no measures expense is required, so only the cast-in-place part contains it, calculated as follows.

$$C_{k}=M_{4a}\times V_{a}$$ (8)

Where *C*_*k*_ refers to the measure expense of the project; *M*_4*a*_ refers to the measure expense of the cast-in-place part a.

Transportation cost

Including the transportation cost of raw materials and prefabricated components, calculated as follows.

$$C_{t}=C_{ct}+C_{pt}$$ (9)

$$C_{t}=\sum_{h=1}^{n}B_{ih}\times V_{d}+\sum_{g=1}^{n}B_{jg}\times V_{j}$$ (10)

Where *C*_*t*_ refers to the transportation cost in the project; *C*_*ct*_, *C*_*pt*_ refers to the transportation cost of raw materials and prefabricated components in the project; *B*_*ih*_, *B*_*jg*_ refers to the cost of transportation used to transport unit volume of *i*^*th*^ raw material and *j*^*th*^ prefabricated component; *V*_*d*_, *V*_*j*_ refers to the volume of *i*^*th*^ raw material and *j*^*th*^ prefabricated component required for the project.

#### Duration

The assembled building adopts the mode of design and construction integration, which saves the construction period under the premise of quality assurance. With the change in the construction period, the time of receiving payment also changes, which accelerates the capital turnover and enhances the intensity of capital supply, and can further promote the development of assembling, so the influence of the construction period is considered in the selection of prefabricated component combination scheme.

In this paper, the theoretical duration of cast-in-place and precast is calculated by the duration saving ratio, where the duration is affected by the number of workers and the volume of work. Assuming constant daily labor consumption, the duration saving ratio of the same unit project under two different construction modes can be calculated using the labor-saving ratio of each component, which is calculated as follows.

$$D_{a}=\frac{P_{C}-P_{p}}{P_{c}}=\frac{D_{c}-D_{p}}{D_{c}}$$ (11)

Where *D*_*a*_ refers to the duration saving ratio of prefabricated component a; *P*_*c*_ and *P*_*p*_ refer to the number of labor required for cast-in-place building components and prefabricated components; *D*_*c*_ and *D*_*p*_ refer to the duration of cast-in-place building components and prefabricated components.

#### Carbon footprint

In 2020, China clearly put forward the goal of "peak carbon dioxide emissions" in 2030 and "carbon neutrality" in 2060. Among them, carbon emissions from buildings are the main cause of the greenhouse effect, and the relevant state departments are actively promoting the research and development of advanced energy-saving and environmental protection technologies, techniques, and equipment, accelerating the upgrading of green buildings in the construction industry, and developing the integration of assembly-type buildings with green buildings and low-carbon buildings as an inevitable trend.

To accurately assess carbon emissions, the calculation boundary must be clearly delineated. According to life cycle theory, a building’s life cycle can be demarcated into four stages. They include the building materials production and transportation stage, construction stage, operation and maintenance stage, and dismantling and recycling stage. The materialization phase includes the building material production and transportation phase and construction phase. In the dismantling and recycling stage, carbon emissions mainly come from energy consumption of building facilities and self-demolition activities, etc., while it does not differ significantly between prefabricated and cast-in-place modes. Thereby, this paper only considers carbon emissions in the materialization stage.

In the production of components, the main work includes mold table cleaning, formwork installation, steel cage installation, embedded parts installation, concrete pouring, curing, and formwork removal. The sources of carbon emissions are classified into two main parts: one is the embedded carbon emissions of construction raw materials. The other part is the carbon emission from equipment operation (including vibrating table and maintenance machine et al.) that consume energy and electricity. In the transportation of prefabricated components to the construction site, carbon emission sources are from vehicles, which mainly consume diesel or gasoline. In the on-site construction phase, the assembly activities are mainly completed by lifting, apart from some cast-in-place work to be carried out on site. Therefore, the main sources of carbon emissions on site are equipment energy consumption and cast-in-situ concrete.

The carbon emission of the prefabricated building not only includes the prefabricated concrete part, and also the cast-in-place part. According to “Building Carbon Emission Calculation Standard”, the carbon emission factor can be obtained. The sum of the carbon emissions, calculated by:

$$C_{f}=C_{1}+C_{2}$$ (12)

Where, *C*_*f*_ refers to the carbon emission of scheme f; *C*_1_ and *C*_2_ refer to the carbon emission of cast-in-place part and prefabricated part.

Cast-in-place part

$$C_{1}=C_{ycl1}+C_{ycl2}$$ (13)

$$C_{\mathrm{ycl}1}=\sum_{i=1}^{n}\frac{L_{1i}}{Z}\times A_{i}\times E_{11}\times E_{x}+\sum_{i=1}^{n}L_{2i}\times A_{i}\times E_{y}$$ (14)

$$C_{\mathrm{ycl}2}=\sum_{i=1}^{n}L_{i}\times B_{i}\times M_{1i}$$ (15)

Where, *C*_*ycl*1_, C_ycl2_ refer to the carbon emissions of cast-in-place components during the raw material transportation phase and construction phase; *L*_*i*_ refers to the consumption of material i; *L*_1*i*_ and *L*_2*i*_ refer to the consumption of concrete and other raw materials respectively; Z refers to the load of the concrete truck; *A*_*i*_ refers to the transportation distance of material i; *E*_11_ refers to the average diesel consumption; *B*_*i*_ refers to the consumption of material i; *M*_1*i*_ refers to the carbon emission factor of material i; *E*_*x*_, *E*_*y*_ refer to the carbon emission factors of diesel and other transport vehicles, respectively.

Prefabricated concrete part

$$C_{2}=C_{yz1}+C_{yz2}+C_{yz3}$$ (16)

$$C_{\mathrm{yz}1}=\sum_{j=1}^{n}L_{j}\times M_{1j}+O_{j}\times M_{2j}$$ (17)

$$C_{yz2}=\sum_{j=1}^{n}N_{j}\times D_{j}\times E_{y}$$ (18)

$$C_{\mathrm{yz}3}=\sum_{j=1}^{n}N_{j}\times P_{j}\times M_{1j}$$ (19)

Where, *C*_yz1_, *C*_*yz*2_, and *C*_yz3_ refer to the carbon emissions from the production, transportation, and assembly of prefabricated components respectively; *L*_*j*_ refers to the energy consumption of production of prefabricated component j; *N*_*j*_ refers to the consumption of prefabricated component j; *D*_*j*_ refers to the distance of transportation of prefabricated component j; *O*_*j*_ refers to the materials consumption of production of prefabricated component j; *P*_*j*_ refers to the mechanical energy consumption used for the assembly of prefabricated unit volume component j; *M*_1*j*_ and *M*_2*j*_ refer to the carbon emission factor; *E*_*y*_ refers to the carbon emission factor of transport vehicles.

### Schemes selection

The comprehensive benefits of assembled buildings are mainly affected by the assembly rate, duration, cost, and environment, which affect each other in the evaluation process. The fuzzy gray correlation projection method combines fuzzy theory, gray system theory, and vector projection, which is a method to analyze the correlation of each index in a comprehensive and specific way, and the main process is:

1. ① Determining the initial matrix C. The attributes of precast component combination scheme A_i_ to indicator B_j_ are recorded as x_ij_, forming an initial matrix C composed of n schemes and m indicators.
2. ② Normalization of the decision matrix to form a standardized matrix *D*= (y_*ij*_)_*n*×*m*_. This includes cost-based and benefits indicators, i.e., the smaller the evaluation index is, or the larger the evaluation index is, the more favorable the prefabricated component assembly solution is.
3. ③ Determining the rational superior and inferior solutions. According to the dimensionless principle, it is known that the larger y_ij_ is the better, and assume that the ideal optimal solution is $A_{0}^{+}$ = {1,1,1,…,1}, and the opposite ideal inferior solution is $A_{0}^{-}$ = {0,0,0,…,0}.
4. ④ Determining the positive and negative weighted gray correlation decision matrix. According to the gray correlation theory, the positive and negative gray correlation coefficients $\varphi_{\mathrm{ij}}^{+}$, $\varphi_{\mathrm{ij}}^{-}$ are calculated, and the positive and negative weighted gray correlation decision matrix $\varphi_{\omega }^{+}$, $\varphi_{\omega }^{-}$ can be obtained by combining the weights calculated from the information entropy.
5. ⑤ Determining the positive and negative weighted gray correlation projection values, $U_{i}^{+}$, $U_{i}^{-}$. The larger $U_{i}^{+}$, the closer the scheme is to the ideal optimal solution, and the larger $U_{i}^{-}$, the closer the scheme is to the ideal inferior solution.
6. ⑥ Calculating the optimal membership degree. The larger *ρ*_i_, the better the scheme, and the alternative scheme with the largest value is the optimal scheme of components combinations.

## Case application

### Case background

**Fig 3:**
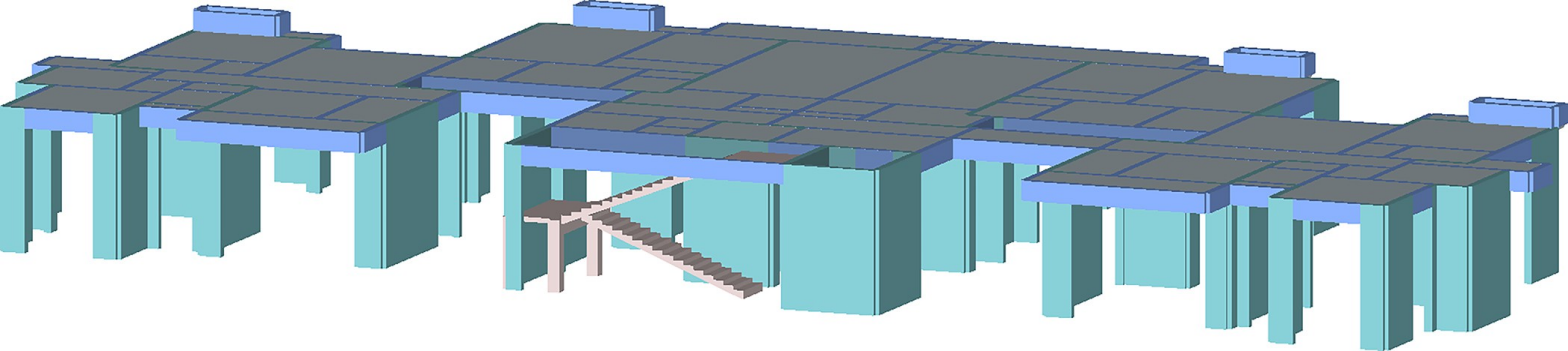
The 3D model of the standard floor.

To verify the application of the precast components combination schemes selection method in precast projects, this paper takes a precast project in Wuhan, Hubei Province, China as an example. The project is a high-rise shear wall structure with 28 floors and 82.4m building height. The 4-28^th^ are standard floors, and the floor area of the standard floors is 941.26xm^2^, and it adopts an assembly-type construction method. The main types of prefabricated components being used include prefabricated beams, prefabricated exterior walls, prefabricated interior walls, prefabricated stairs, prefabricated balconies, prefabricated air conditioning panels, and prefabricated floor slabs. According to the architectural drawings and component details of this project, the 3D model of the standard floor was drawn by PKPM-PC software as shown in Fig 3. The concrete volume and projected area of the vertical and horizontal precast components were obtained, as shown in Tables 2 and 3.

**Table 2:** The concrete volume of vertical precast components.

	The concrete volume of precast components(m^3^)		Total concrete volume(m^3^)
	Prefabricated exterior walls	Prefabricated interior walls	
Standard floor	51.03	15.028	108.116
Whole building	1224.72	360.672	3027.248

**Table 3:** The projected area of horizontal precast components.

	The projected area of horizontal precast components(m^2^)					Total area(m^2^)
	Prefabricated beams	Prefabricated floor slabs	Prefabricated stairs	Prefabricated balconies	Prefabricated air conditioning panels	
Standard floor	48.997	292.966	20.01	54.911	15.356	465.345
Whole building	1224.9	7324.15	500.25	1372.775	383.9	13029.66

### Determination of the scheme of prefabricated components

The main structure of the assembled building accounts for a large proportion, which directly affects the assembly rate of the project. Meanwhile, the load-bearing walls play a larger role compared with other minor force-bearing elements (stairs, balcony slabs, etc.). Consequently, the selection of prefabricated components needs to be analyzed by combining the proportion of components and process installation to determine the selection order of prefabricated components. The study was based on the three most widely used members, namely stairs, balcony slabs, and air conditioning panels. The prefabricated components combination scheme was carried out by the principle of permutation and combination, and 16 prefabricated component combination schemes were obtained, as shown in the first three columns of Table 4.

**Table 4:** The combination scheme of prefabricated components.

Quantity of prefabricated components in the combination scheme	Number	Schemes of prefabricated components^a^	Cast-in-place components	Assembly rate
**3**	A	1+2+3	4+5+6+7	——
**4**	B	1+2+3+4	5+6+7	——
	C	1+2+3+5	4+6+7	58.6%
	D	1+2+3+6	4+5+7	——
	E	1+2+3+7	4+5+6	——
**5**	F	1+2+3+4+5	6+7	68.2%
	G	1+2+3+4+6	5+7	54.5%
	H	1+2+3+4+7	5+6	——
	I	1+2+3+5+6	4+7	88.9%
	J	1+2+3+5+7	4+6	58.6%
	K	1+2+3+6+7	4+5	——
**6**	L	1+2+3+4+5+6	7	94.5%
	M	1+2+3+4+5+7	6	68.2%
	N	1+2+3+4+6+7	5	54.5%
	O	1+2+3+5+6+7	4	88.9%
**7**	P	1+2+3+4+5+6+7	——	94.5%

^a^1: prefabricated stairs; 2: prefabricated balconies; 3: prefabricated air conditioning panels; 4: prefabricated interior walls; 5: prefabricated exterior walls; 6: prefabricated floor slabs; 7: prefabricated beams.

### Indicator calculation

Assembly rate

According to the “Standard for assessment of prefabricated building”, the scores of the above 16 prefabricated components combination schemes are calculated, shown in the last column of Table 2, and the combination schemes that meet the requirements are selected. The main structure score of A, B, E, and H is less than 20 points, and the score of the enclosure wall and internal partition wall of D and K is less than the minimum scoring standard, so A, B, E, H, D, and K do not meet the requirements and thus are not considered. The remaining schemes contain schemes with the same assembly rate, and the difference lies in whether prefabricated beams are used or not. For the convenience of calculation, C, F, G, I, L, and P combination schemes are selected as the evaluation objects in this paper.

Cost

Based on the unit costs of cast-in-place and prefabricated components at different stages and the corresponding engineering quantity, the costs of different combination schemes are calculated. The data are obtained from the “Prefabricated construction consumption quota” and “Housing construction and decoration engineering consumption quota”. According to the survey, the distance of the project from the prefabricated components factory is 60km, the single transportation of components is about 25t, and the transportation cost of prefabricated components is 128 RMB/m^3^ considering the secondary transportation cost in the site, and the single cost of different stages is calculated, as shown in Table 5. The total cost of construction of the standard floor for different schemes was calculated by combining different quantities of work, as shown in Table 6.

**Table 5:** Single-party cost for different phases(unit: Yuan).

No.	Prefabricated component	Labor cost	Material cost	Machinery cost	Transportation fee	Sum
**1**	Exterior walls	631.0	1256.7	1261.2	128	3276.9
**2**	Interior walls	613.9	1326.8	1272.1	128	3326.8
**3**	Beams	512.9	1655.6	1746.4	128	4042.9
**4**	Slabs	604.8	1288.1	1028.5	128	3049.4
**5**	Stairs	631.0	1121.8	1146.9	128	3027.7
**6**	Balconies	601.1	1033.2	1146.9	128	3309.2
**7**	Air conditioning panels	624.8	1148.3	1025.5	128	2926.6

**Table 6:** Total cost of prefabricated components combination schemes.

Scheme	Assembly rate	Total cost(Unit: yuan)	Scheme	Assembly rate	Total cost(Unit: yuan)
**C**	58.6%	427,052.33	I	88.9%	548,454.75
**F**	68.2%	529,687.28	L	94.5%	558,027.46
**G**	54.5%	504,027.17	P	94.5%	562,772.22

Duration

According to the “Housing construction and decoration engineering consumption quota” and “Prefabricated construction consumption quota”, the construction period saving of components and the duration saving of prefabricated components combination schemes are calculated as shown in Tables 7 and 8.

**Table 7:** Prefabricated components duration savings (unit: Workday).

	Beams	Exterior walls	Interior walls	Slabs	Balconies	Stairs	Air conditioning panels
**Prefabricated**	2.28	2.15	1.6	3.04	0.64	1.53	0.63
**Cast-in-place**	6	5	4	4	2	3	1
**Duration savings**	3.72	2.85	2.4	0.96	1.36	1.47	0.37

**Table 8:** Duration savings for the combined schemes.

Scheme	Assembly rate	Duration savings / Workday
**C**	58.6%	6.05
**F**	68.2%	8.45
**G**	54.5%	6.56
**I**	88.9%	7.01
**L**	94.5%	9.41
**P**	94.5%	13.13

Carbon footprint

Through the statistical analysis of the research results of prefabricated components factories, the consumption of materials and equipment per unit volume in the prefabricated components production stage was obtained as shown in Tables 9 and 10.

**Table 9:** Raw material consumption in the production stage of prefabricated components per unit volume.

Prefabricated components	Exterior walls	Interior walls	Beams	Slabs	Stairs	Balconies	Air conditioning panels
Concrete (m^3^)	0.97	0.97	0.96	0.98	0.99	0.97	0.99
Rebar (kg)	130.04	100.38	258.43	195.08	148.31	136.25	104.27

**Table 10:** Energy consumption of mechanical equipment in the production stage of prefabricated components per unit volume.

Prefabricated components	Exterior walls	Interior walls	Beams	Slabs	Stairs	Balconies	Air conditioning panels
**Electricity (kwh/m^3^)**	15	14	8	12	11	11	7
**Diesel volume (kg/m^3^)**	0.63	100.38	0.63	0.63	0.63	0.63	0.63

Prefabricated components are transported by heavy diesel truck (30t). The transportation distance is about 60km, and the round-trip coefficient is 1.67, so the carbon emission per unit volume of precast components in the transportation stage is 19.54kgCO_2_eq/m^3^. In addition to the prefabricated component transportation carbon emissions, the carbon emission per unit volume of concrete transportation is 34.08 kgCO_2_eq/m^3^, and the carbon emission per unit volume of rebar is 6.55 kgCO_2_eq/m^3^.

The cranes are mainly used in the on-site construction stage, and the relevant data are obtained from the “Prefabricated construction consumption quota” and the carbon emission of the cast-in-place part in the construction stage is 613.13 kgCO_2_eq/m^3^. The carbon emissions of different prefabricated components per unit volume are summarized and shown in Table 11.

**Table 11:** Carbon emissions per unit volume of prefabricated components(unit: kgCO_2_eq/m^3^).

No.	Prefabricated components	Production	Transportation	Construction	Sum
**1**	Exterior walls	609.38	19.54	10.99	639.91
**2**	Interior walls	537.78	19.54	10.99	568.31
**3**	Beams	908.28	19.54	12.67	940.49
**4**	Slabs	765.63	19.54	32.98	818.15
**5**	Stairs	656.09	19.54	7.65	683.28
**6**	Balconies	621.37	19.54	19.36	660.27
**7**	Air conditioning panels	549.97	19.54	26.53	594.04

According to Table 11, the carbon emissions of different prefabricated components combination schemes are obtained by combining with the engineering quantities, as shown in Table 12.

**Table 12:** Carbon emissions of different combination schemes(unit: tCO_2_eq).

Schemes	Assembly rate	Total Carbon Emissions	Schemes	Assembly rate	Total Carbon Emissions
**C**	58.6%	139.98	I	88.9%	125.75
**F**	68.2%	129.26	L	94.5%	120.84
**G**	54.5%	130.39	P	94.5%	120.12

### Optimized selection options

Determine the initial matrix

Based on the data in the actual case, where assembly rate and duration are benefit indicators, and cost and carbon footprint are cost indicators, the initial decision matrix was obtained, as shown in Table 13.

**Table 13:** The initial decision matrix.

	Assembly rate	Cost	Duration	Carbon footprint
**C**	58.60	42.71	6.05	139.98
**F**	68.20	50.23	8.45	129.26
**G**	54.50	48.40	6.56	130.39
**I**	88.90	54.84	7.01	125.75
**L**	94.50	55.80	9.41	120.8
**P**	94.50	56.28	13.13	120.12

Determine indicator weights and gray correlation projection weights

According to the nature of different indicators, the initial matrix was standardized and the entropy weight method was applied to calculate the weights of each indicator, so as to obtain the gray correlation projection weights of each indicator, as shown in Table 14.


$$x_{\mathrm{ij}}^{'}=(\begin{array}{cccc} 0.90 & 0.14 & 0.09 & 0.09\\ 0.99 & 0.17 & 0.12 & 0.12\\ 0.78 & 0.13 & 0.09 & 0.09\\ 1.00 & 0.13 & 0.08 & 0.08\\ 1.00 & 0.17 & 0.10 & 0.11\\ 0.81 & 0.23 & 0.11 & 0.11 \end{array})$$ (20)

**Table 14:** Indicators weights and gray correlation projection weights.

Indicator	Weights	Gray correlation projection weights
**Assembly rate**	0.26	0.13
**Cost**	0.30	0.18
**Duration**	0.22	0.10
**Carbon footprint**	0.22	0.10

Schemes Selection

The positive and negative correlation matrices(*φ*^+^, *φ*^−^) are calculated to provide the positive gray correlation projection values ($U_{i}^{+}$), negative gray correlation projection values ($U_{i}^{-}$), and the optimal membership degree (*ρ*_i_) of the different prefabricated component combinations, as shown in Table 15.


$$\varphi^{+}=(\begin{array}{cccc} 0.36 & 1.00 & 0.33 & 0.33\\ 0.43 & 0.40 & 0.43 & 0.52\\ 0.33 & 0.47 & 0.35 & 0.49\\ 0.78 & 0.36 & 0.37 & 0.64\\ 1.00 & 0.34 & 0.49 & 0.93\\ 1.00 & 0.33 & 1.00 & 1.00 \end{array})\;\varphi^{-}=(\begin{array}{cccc} 0.83 & 0.33 & 1.00 & 1.00\\ 0.59 & 0.67 & 0.60 & 0.48\\ 1.00 & 0.54 & 0.87 & 0.51\\ 0.37 & 0.83 & 0.79 & 0.41\\ 0.33 & 0.94 & 0.51 & 0.34\\ 0.33 & 1.00 & 0.33 & 0.33 \end{array})$$ (21)

**Table 15:** The positive and negative weighted gray correlation projection values and the optimal membership degree.

Schemes	$U_{i}^{+}$	$U_{i}^{-}$	*ρ*_i_
**C**	0.29	0.36	0.30
**F**	0.22	0.30	0.33
**G**	0.21	0.36	0.29
**I**	0.27	0.31	0.39
**L**	0.39	0.29	0.77
**P**	0.33	0.29	0.60

## Results and discussions

### Results

**Fig 4:**
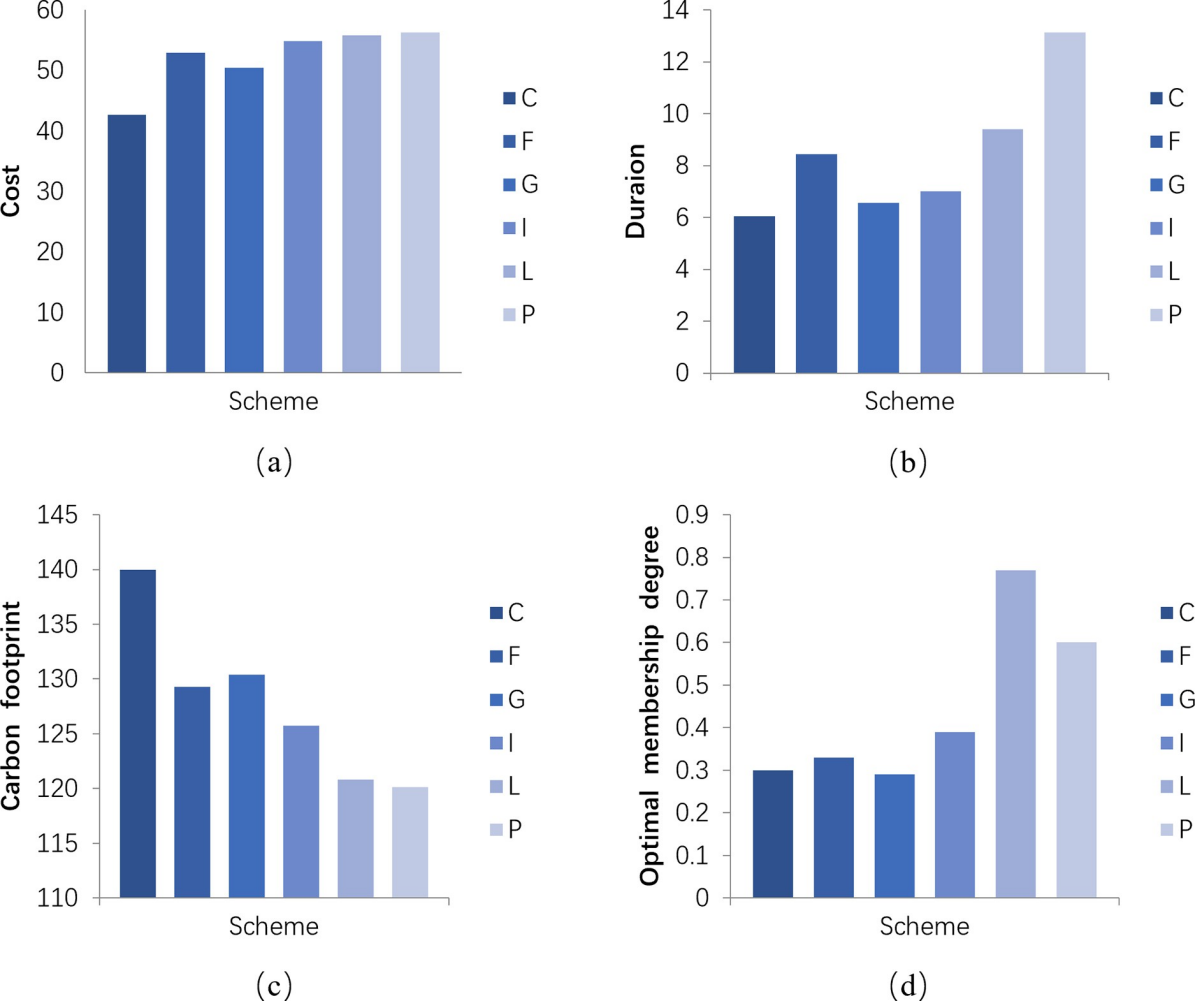
Analysis of indicators of different schemes. (a) Costs under different schemes(million),(b) Duration savings under different schemes (Workday),(c) Carbon footprint under different schemes (tCO_2_eq),(d) Optimal membership degree under different schemes.

According to the results in Fig 4(A)–4(C), the cost is positively correlated with the assembly rate within a certain range of assembly rates. With the increase in the assembly rate, the total cost under the assembly construction mode tends to increase, and the assembly rate at the lowest cost is 58.6%; in terms of duration, as the assembly rate increases, the duration saving ratio also increases, and the overall construction duration shows a decreasing trend, with the maximum duration saving when the assembly rate is 94.5%; for carbon emissions, it decreases with the increase of assembly rate and the least carbon emission with 94.5% of assembly rate. From Fig 4(D), we can see that the magnitude relationship of the optimal membership degree is $\rho_{L}>\rho_{P}>\rho_{I}>\rho_{F}>\rho_{C}>\rho_{G}$, which gives the solution L as the optimal solution.

Fig 4 shows the trend of the change of each indicator with increasing assembly rate, where there are different cases generated. (1)The effect of assembly rate on cost. The assembly rates of schemes L and P are both 94.5%, but the costs are RMB 558,000 and RMB 562,800, respectively, which are due to the different types and quantities of prefabricated components. (2) With the same assembly rate, the number of prefabricated components has a different impact on the construction period. As in schemes L and P, option P has a larger number of prefabricated components, resulting in more savings in construction time. (3) In the case of the same number of prefabricated components, the larger the assembly rate, the longer the construction period. For example, schemes F and I, which is due to the different precast component composition schemes with different degrees of influence.

In addition, according to Tables 6, 8, and 12, it can be seen that the prefabricated components are selected in different orders considering from different perspectives. (1) From the cost, the order is air conditioning slabs, stairs, floor slabs, external walls, balconies, internal walls, and beams. (2) From the duration of work, the order is balconies, beams, interior walls, exterior walls, stairs, air conditioning panels, and floor slabs. (3)From the carbon footprint, the order is interior walls, air conditioning panels, exterior walls, balconies, stairs, floor slabs, and beams.

## Discussions

The applicability and feasibility of the method used in this paper are verified through empirical analysis, and the selection of prefabricated components scheme for the main structure of the assembled building with a shear wall structure can be effectively solved by this method. It provides a reference for the construction unit’s pre-decision and avoids blind selection.

Among the four indicators studied in this paper, are assembly rate, cost, duration, and carbon footprint, according to weight division, the cost has the greatest influence on the prefabricated component combination scheme, followed by assembly rate, and finally duration and carbon footprint. In order to improve the superiority of the combination scheme, several suggestions are suggested according to the degree of impact of the indicators.

(1) In the early stage of design, according to the principle of "fewer specifications and more combinations", realize the standardization of design, improve the turnover times of molds, reduce amortization expenses, and effectively reduce the production cost of prefabricated components. (2) The scheme with a higher assembly rate is prioritized over the scheme with a lower assembly rate. (3) The way to increase the assembly rate includes not only increasing the types of prefabricated components but also the option of integral toilets, integral kitchens, etc. (4) Nevertheless, in China, the prefabricated buildings are in the initial stage, the related construction and management techniques are not mature enough. It is advisable to use materials with small carbon emission factors and also to reduce material waste by improving processes or construction methods, thus reducing carbon emissions.

Despite the realization of the study objectives, the method still has limitations. First of all, the comprehensive benefits analysis dimension selected in this study is only a part of it, and there are many other factors, such as social benefits, safety benefits, and some qualitative factors that cannot be analyzed quantitatively. Second, some of the assumptions in the quantification process are more demanding. Third, in the carbon emission calculation, different carbon emission factors will make the carbon emission result different, so the carbon emission factor should be selected according to the area where the case building is located. Fourth, the prefabricated component combination schemes in this paper only consider the prefabrication of the main structural components and do not consider the prefabrication of toilets, kitchens, etc.

## Conclusion

In this paper, using the prefabricated components combination schemes as the research object. Based on the comprehensive benefits analysis, the prefabricated component combination schemes are compared by the fuzzy gray correlation projection method to assist decision-makers in selecting the optimal scheme. Based on the results and discussions, the following conclusions can be drawn.

1. Increasing the proportion of prefabricated components in assembled building projects is critical to the comprehensive benefits of assembled buildings. By changing the type and quantity of prefabricated components, thus the combination scheme of prefabricated components with the optimal comprehensive benefit is obtained.
2. The selection order of prefabricated components under a single index is different. Due to the different prefabricated components have different degrees of influence on it.
3. Without affecting the quality of the project, the horizontal components are generally selected first, and then the vertical components are added when the assembly rate requirement is not met. Selecting the appropriate assembly rate can improve the buildability of the assembled building.

The method used in this study has the advantages of high sensitivity, simple operation, and easy implementation, and has the value of promotion and application, which can provide a reasonable and effective reference for the investors and real estate developers of prefabricated buildings when making scheme comparisons. Furthermore, the research and analysis presented in this article will aid researchers and practitioners in better addressing the complex and challenging issues in this field in the future, as well as provide new insights and methods for solving practical problems. It also can offer guidance for analyzing quantitative data in the evaluation of prefabricated buildings, laying a foundation for future research on prefabricated buildings.

Further research and discovery of advanced methods are needed in the future to enable a more efficient and accurate selection of prefabricated combination schemes. In addition, other prefabricated components, integrated toilets, integrated kitchens, and integrated stairs could be added to improve the research system.
